# Induction of bulb organogenesis in *in vitro* cultures of tarda tulip (*Tulipa tarda* Stapf.) from seed-derived explants

**DOI:** 10.1007/s11627-014-9641-1

**Published:** 2014-10-01

**Authors:** Małgorzata Maślanka, Anna Bach

**Affiliations:** Department of Ornamental Plants, University of Agriculture in Kraków, Al. 29 Listopada 54, 31-425 Kraków, Poland

**Keywords:** *Tulipa tarda*, Bulbing, 6-Benzyl-aminopurine, Abscisic acid

## Abstract

A protocol for obtaining bulbs via *in vitro* organogenesis was developed for tarda tulip (*Tulipa tarda* Stapf). Scale explants were obtained from bulbs formed at the base of seedlings or from adventitious bulbs that developed from callus tissue forming on stolons or on germinating seeds. Some explants were subjected to chilling at 5°C for 12 wk. The culture media contained 3 or 6% sucrose and was supplemented with either no growth regulators, either 0.5 μM 6-benzyl-aminopurine (BAP) or 18.9 or 94.6 μM abscisic acid (ABA). Cultures were maintained in the dark at 20°C. Callus tissue developed mainly on media without growth regulators or with BAP. Callus was formed from up to 96% of explants derived from non-chilled adventitious bulbs that were treated with 3% sucrose and 0.5 μM BAP. Less callus was formed from chilled explants compared with non-chilled explants. Newly formed adventitious bulbs appeared on the explants via direct and indirect organogenesis. The media with BAP promoted the formation of adventitious bulbs at a rate of 56–92% from non-chilled explants, whereas a maximum rate of 36% was observed from chilled explants. ABA inhibited the induction of adventitious bulbs and callus. The adventitious bulbs obtained in these experiments contained a meristem, which was evidence that they had developed properly.

## Introduction

Tulips are the most economically important bulbous ornamental plants and have been among the top species produced for cut flowers and bedding for many years (Taghi *et al.* 2007). Traditionally propagated plants have a long juvenile period of 4–5 yr (Eijk *et al.* 1991; Rees 1992; Custers *et al.* 1997), a low reproduction rate, and it takes 25 yr to introduce a new cultivar into production (De Hertogh and Le Nard 1993). The cost of cultivating tulips is therefore high.

*Tulipa tarda* Stapf. is a member of the botanical tulip group that is a native of Central Asia and is particularly valuable. Due to its low height, a multi-flowered stem (up to six flowers) and the capacity to remain in one place for several years, it is useful for urban green areas unsuitable for other species of tulips (Botschantzeva 1982). The use of biotechnology techniques makes it possible to shorten the time needed to cultivate tulips and to increase their rate of reproduction. However, the laboratory methods for the propagation of tulips remain low yielding (Wilmink *et al.* 1995; Podwyszyńska and Marasek 2003; Ptak and Bach 2007; Maślanka and Bach 2010). New, more efficient means of *in vitro* tulip propagation are needed.

*In vitro* micropropagation of bulbous plants can yield 1,000 descendant bulbs in 1.6 yr from one bulb, which takes about 16 yr under natural conditions (Rees and Hanks 1979). Organogenesis is one method of *in vitro* plant propagation (Hulscher *et al.* 1992; Wilmink *et al.* 1995; Podwyszyńska and Marasek 2003; Ghaffor *et al.* 2004). Organogenic cultures can be induced from explants obtained from any living part of the plant, including seeds (Niimi 1978; Boeken and Gutterman 1990; Famelaer *et al.* 2000; Rouhi *et al.* 2010).

The formation of tulip bulbs is influenced by both chemical and physical characteristics of the culture environment and can be stimulated with appropriate cytokinin treatment, an increased level of sucrose and by chilling the explants. Especially important during *in vitro* organogenesis or “bulbing” is abscisic acid (ABA), which affects the accumulation of storage proteins and lipids (Seo and Koshiba 2002).

Although conditions suitable for bulb formation *in vitro* have been preliminarily defined for tulip, this process remains inefficient and unsuitable for obtaining a sufficient number of bulbs in a short time to enable their mass production. In this study, we have focused on tarda tulip in order to develop a more efficient bulbing protocol. The effects of explant chilling, sucrose concentration and 6-benzyl-aminopurine (BAP) and ABA treatments were investigated for their effects on obtaining properly developed adventitious bulbs.

## Materials and Methods

### Plant material. Seed germination.

*T. tarda* Stapf. (common name tarda or late tulip) seed was harvested in July, 2009. In October, seeds were surface-disinfected with 70% ethanol for 1 min followed by immersion in a 15% solution of Domestos (Unilever, Poland). The seeds were then rinsed three times with sterile water. Disinfected seeds were plated on nutrient media containing either full or half strength (Murashige and Skoog 1962) with 3% sucrose. All media were solidified with 0.5% Lab-agar (Biocorp, Poland), and the pH of all media was adjusted to 5.8 before autoclaving. Each seed was placed individually in a 50-ml glass tube containing 10-ml medium and chilled for 10 wk at 5°C in darkness. Cultures were then placed at 20 or 25°C under illumination with daylight fluorescent lamps (30 μmol m^−2^ s^−1^) with a 16-h photoperiod. Germination was observed 6 mo after the end of the chilling treatment. For the next 4 mo, seed germination characteristics were assessed. Properly germinating seeds developed bulbs on the base of the seedlings, as compared to seeds that formed callus from which many smaller adventitious bulbs developed. All bulbs were used as explants for further experiments.

### *Induction of callus tissue and adventitious bulbs on scale explants.*

Scale explants (Fig. 2*a*, *b*), consisting of 4–5-mm length pieces of bulbs cut lengthwise, were cultured in Petri dishes (90 × 25 mm) on 25-ml MS solid medium (0.5% Lab-agar), containing 0.5 μM BAP, 18.9 or 94.6 μM ABA, 3 or 6% sucrose, with a pH of 5.8. After 1 wk, explants were transferred to a fresh medium as described above but without ABA. The cultures were maintained in the dark at 20°C for 12 wk to allow the formation of adventitious bulbs. Subsequently, the frequencies of the formation of callus tissue and adventitious bulbs (expressed as a percentage) and the number of bulbs per replicate were assessed. The experiment was repeated with explants that had been subjected to chilling at 5°C for 12 wk.

### *Dynamics of the adventitious bulbs and callus tissue formation.*

Undifferentiated callus tissue from non-chilled bulbs was used as the explant in this experiment. Portions of callus (5 × 0.2 g; 1 g total) were transferred to Petri dishes (90 × 25 mm) containing 25-ml media of the same formulation as that on which the callus had formed. The media consisted of MS mineral salts, with either 3 or 6% sucrose. Half were supplemented also with 0.5 μM BAP. All media were adjusted to pH 5.8 and solidified with 0.5% Lab-agar. The cultures continued to be maintained in the dark at 20°C. After 12 wk, the proliferation of callus, or relative growth rate (RGR = increase in callus weight during the test period/initial weight), the formation of adventitious bulbs (expressed as a percentage), and the number of bulbs per replicate were assessed.

### *Anatomical observations.*

Adventitious bulbs were examined using a binocular microscope. Histological studies were carried out on samples imbedded in paraffin after fixation in ethanol/acetic acid (3:1, *v*/*v*) (Gerlach 1969). Serial sections were cut to 10 μm with a microtome (RM 2145, Leica, Germany) and were stained with safranin and fast green (Jensen 1962).

### *Statistical analysis.*

The experiment with seeds was performed with five replicates of 7–8 seeds each. The experiment on the induction and characteristics of bulb formation of bulbs was performed with five replicates of five scale explants each. The results were analysed using the two-factorial method of analysis of variance. Comparison of mean values was made with Tukey’s test at a significance level of *P* ≤ 0.05 using the STATISTICA 9 software package.

## Results

### *Seed germination.*

Germination of cultured seeds was observed after 6 mo and continued over a period of 4 mo. Initially, the radicle developed followed by the hypocotyl. Subsequently, the dropper and cotyledon developed, but some seeds formed callus (data not shown). Properly germinating seeds developed a bulb on the seedling base, and many smaller adventitious bulbs developed from the callus.

**Figure 1:**
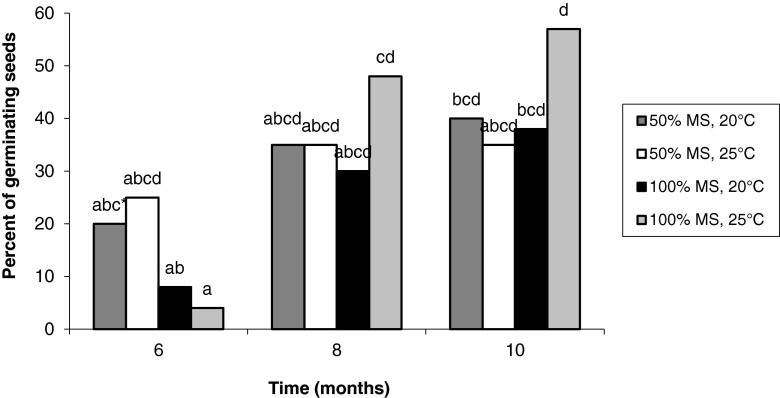
Effect of cultivation time, MS concentration and temperature on seed germination.

A higher percentage of germinating seeds was observed on 50 versus 100% MS medium after 6 mo of culture. After 10 mo, the highest germination percentage was observed from seeds cultured on 100% MS and 25°C (Fig. 1).

### *Induction of callus tissue and adventitious bulbs on scale explants.*

**Figure 2:**
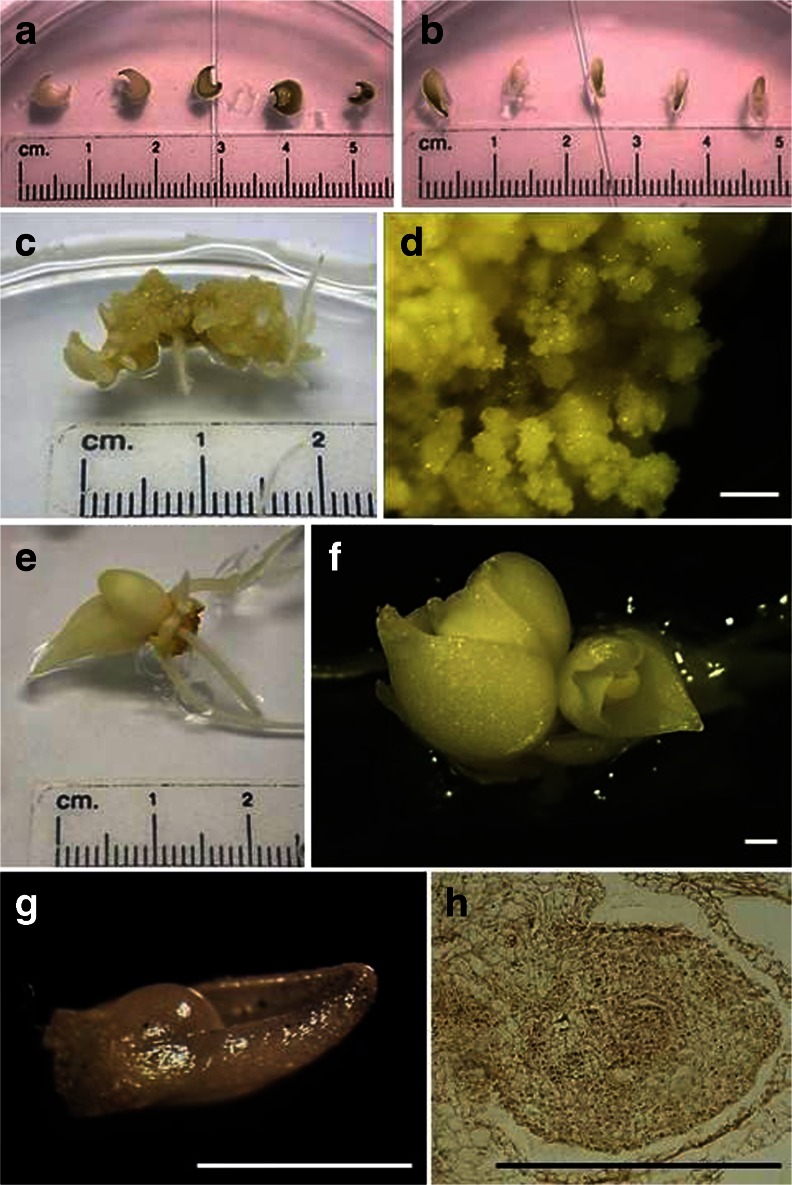
Explant types of *T. tarda* used (*a*) bulb pieces from seedlings, (*b*) bulb pieces from adventitious bulbs. Induction of (*c*, *d*) callus, (*e*, *f*) adventitious bulbs, (*g*) meristem isolated from adventitious bulb, (*h*) anatomical cross-section of a bulb. *Bars* = 1,000 μm.

Callus tissue that was initially transparent, and which then became yellow, (Fig. 2*c*, *d*) gradually appeared on the explants and was present mainly on explants cultured on medium without growth regulators or medium supplemented with BAP (Tables 1, 2, 3 and 4). After 12 wk, up to 96% explants derived from adventitious bulbs formed callus when cultures with 3% sucrose and 0.5 μM BAP as compared to other treatments (Table 3). BAP treatment significantly enhanced the frequency of callus induction, but only from non-chilled explants derived from adventitious bulbs, and only on the media containing the 6% sucrose (Tables 3 and 4). Chilled explants (Tables 2 and 4) formed callus to a lesser extent compared to non-chilled explants (Tables 1 and 3).

**Table 1:** Effect of medium composition and time on the induction of callus and bulbs from non-chilled seedling-derived bulbs

Culture medium							
Sucrose [%]	Growth regulators [μM]	Explants forming callus [%]			Explants forming bulbs [%]		
		4 wk	8 wk	12 wk	4 wk	8 wk	12 wk
3	–	0.0 a*	44.0 bc	72.0 d	56.0 efg	72.0 efgh	92.0 h
	0.5 BAP	0.0 a	52.0 cd	68.0 d	44.0 cdef	60.0 efg	72.0 efgh
	18.9 ABA	0.0 a	4.0 a	24.0 ab	16.0 abcd	16.0 abcd	56.0 efg
	94.6 ABA	0.0 a	0.0 a	0.0 a	0.0 a	8.0 abc	32.0 bcde
6	–	0.0 a	44.0 bc	56.0 cd	48.0 def	52.0 def	88.0 gh
	0.5 BAP	0.0 a	68.0 d	72.0 d	56.0 efg	60.0 efg	84.0 fgh
	18.9 ABA	0.0 a	4.0 a	20.0 ab	0.0 a	4.0 ab	24.0 abcd
	94.6 ABA	0.0 a	0.0 a	0.0 a	0.0 a	0.0 a	24.0 abcd

*Mean values followed by the same letters in columns do not differ at the significance level of *P* ≤ 0.05

**Table 2:** Effect of medium composition and time on the induction of callus and bulbs from chilled seedling-derived bulbs

Culture medium							
Sucrose [%]	Growth regulators [μM]	Explants forming callus [%]			Explants forming bulbs [%]		
		4 wk	8 wk	12 wk	4 wk	8 wk	12 wk
3	–	0.0 a*	16.0 abc	16.0 abc	20.0 bcde	20.0 bcde	28.0 de
	0.5 BAP	4.0 a	20.0 abcd	32.0 cde	12.0 abcd	20.0 bcde	36.0 e
	18.9 ABA	0.0 a	0.0 a	4.0 a	12.0 abcd	16.0 abcd	20.0 bcde
	94.6 ABA	0.0 a	0.0 a	0.0 a	0.0 a	0.0 a	0.0 a
6	–	0.0 a	20.0 abcd	40.0 e	4.0 ab	4.0 ab	24.0 cde
	0.5 BAP	0.0 a	12.0 ab	36.0 de	0.0 a	12.0 abcd	24.0 cde
	18.9 ABA	8.0 ab	8.0 ab	8.0 ab	4.0 ab	8.0 abc	12.0 abcd
	94.6 ABA	0.0 a	0.0 a	0.0 a	0.0 a	0.0 a	4.0 ab

*Mean values followed by the same letters in columns do not differ at the significance level of *P* ≤ 0.05

**Table 3:** Effect of medium composition and time on the induction of callus and bulbs from non-chilled adventitious bulbs

Culture medium							
Sucrose [%]	Growth regulators [μM]	Explants forming callus [%]			Explants forming bulbs [%]		
		4 wk	8 wk	12 wk	4 wk	8 wk	12 wk
3	–	0.0 a*	52.0 de	64.0 def	20.0 ab	52.0 bcd	68.0 d
	0.5 BAP	8.0 ab	88.0 f	96.0 f	0.0 a	52.0 bcd	72.0 d
	18.9 ABA	0.0 a	0.0 a	0.0 a	0.0 a	0.0 a	0.0 a
	94.6 ABA	0.0 a	0.0 a	8.0 ab	0.0 a	0.0 a	0.0 a
6	–	0.0 a	16 abc	40.0 bcd	20.0 ab	52.0 bcd	56.0 cd
	0.5 BAP	12.0 abc	64.0 def	76.0 ef	24.0 abc	64.0 d	80.0 d
	18.9 ABA	0.0 a	0.0 a	0.0 a	0.0 a	0.0 a	0.0 a
	94.6 ABA	0.0 a	0.0 a	0.0 a	0.0 a	0.0 a	0.0 a

*Mean values followed by the same letters in columns do not differ at the significance level of α *P* ≤ 0.05

**Table 4:** Effect of medium composition and time on the induction of callus and bulbs from chilled adventitious bulbs

Culture medium							
Sucrose [%]	Growth regulators [μM]	Explants forming callus [%]			Explants forming bulbs [%]		
		4 wk	8 wk	12 wk	4 wk	8 wk	12 wk
3	–	0.0 a*	12.0 ab	32.0 c	0.0 a	12.0 ab	28.0 bc
	0.5 BAP	0.0 a	16.0 b	38.0 c	0.0 a	16.0 abc	32.0 c
	18.9 ABA	0.0 a	0.0 a	4.0 ab	0.0 a	0.0 a	8.0 a
	94.6 ABA	0.0 a	0.0 a	0.0 a	0.0 a	0.0 a	4.0 a
6	–	0.0 a	0.0 a	0.0 a	8.0 a	12.0 ab	12.0 ab
	0.5 BAP	8.0 ab	12.0 ab	32.0 c	4.0 a	8.0 a	18.0 abc
	18.9 ABA	0.0 a	4.0 ab	4.0 ab	8.0 a	8.0 a	8.0 a
	94.6 ABA	0.0 a	0.0 a	0.0 a	0.0 a	0.0 a	0.0 a

*Mean values followed by the same letters in columns do not differ at the significance level of *P* ≤ 0.05

One week of exposure to ABA, especially at the higher concentration, inhibited the induction of callus, regardless of sucrose concentration and explant type (Tables 1, 2, 3 and 4).

Adventitious bulbs (Fig. 2*e*, *f*) developed on the explants via direct and indirect (via callus) organogenesis within 4 wk of culture. The percentage of explants producing adventitious bulbs increased steadily up to 12 wk of culture. The media containing only sucrose and BAP promoted the formation of adventitious bulb at a frequency of 56–92% from non-chilled explants (Tables 1 and 3), as compared to a maximum frequency of 36% from chilled explants (Tables 2 and 4). Short roots were observed at the base of the adventitious bulbs.

ABA had a negative effect on the induction of adventitious bulbs (Tables 1, 2, 3 and 4). This was particularly evident in the case of the explants derived from from non-chilled adventitious bulbs, from which the formation of bulbs was not observed (Table 3).

Taking into consideration the effect of the type of explant on the induction of callus, significant differences between means were observed only between the non-chilled and chilled adventitious bulbs treated with BAP. The formation of callus from non-chilled adventitious bulbs was more frequent (76 and 96% of explants) than from chilled adventitious bulbs (32 and 38% of explants) (Table 5).

**Table 5:** Effect of explant type and medium composition on the induction of callus, adventitious bulbs and the number of these bulbs after 12 wk of cultivation

Explant	Culture medium		Feature assessed		
	Sucrose [%]	Growth regulators [μM]	Explants forming callus [%]	Explants forming bulbs [%]	Number of bulbs
Non-chilled seedling-derived bulb	3	–	72.0 def*	92.0 e	15.2 def
		0.5 BAP	68.0 def	72.0 de	16.2 def
		18.9 ABA	24.0 abc	56.0 bcde	6.0 abcd
		94.6 ABA	0.0 a	32.0 abcd	1.6 ab
	6	–	56.0 bcdef	88.0 e	13.2 cdef
		0.5 BAP	72.0 def	84.0 e	14,2 def
		18.9 ABA	20.0 ab	24.0 ab	1.6 ab
		94.6 ABA	0.0 a	24.0 ab	1.2 ab
Chilled seedling-derived bulb	3	–	16.0 ab	28.0 abc	5.8 abcd
		0.5 BAP	32.0 abcd	36.0 abcd	5.8 abcd
		18.9 ABA	4.0 a	20.0 ab	2.6 abc
		94.6 ABA	0.0 a	0.0 a	0.0 a
	6	–	40.0 abcde	24.0 ab	2.6 abc
		0.5 BAP	36.0 abcde	24.0 ab	2.8 abc
		18.9 ABA	8.0 a	12.0 a	2.6 abc
		94.6 ABA	0.0 a	4.0 a	0.2 ab
Non-chilled Adventitious bulb	3	–	64.0 cdef	68.0 cde	9.2 abcde
		0.5 BAP	96.0 f	72.0 de	18.8 ef
		18.9 ABA	0.0 a	0.0 a	0.0 a
		94.6 ABA	8.0 a	0.0 a	0.0 a
	6	–	40.0 abcde	56.0 bcde	13.8 def
		0.5 BAP	76.0 ef	80.0 e	14.0 def
		18.9 ABA	0.0 a	0.0 a	0.0 a
		94.6 ABA	0.0 a	0.0 a	0.0 a
Chilled adventitious bulb	3	–	32.0 abcd	28.0 abc	10.8 bcdef
		0.5 BAP	38.0 abcde	32.0 abcd	6.2 abcd
		18.9 ABA	4.0 a	8.0 a	2.2 ab
		94.6 ABA	0.0 a	4.0 a	0.4 ab
	6	–	0.0 a	12.0 a	1.8 ab
		0.5 BAP	32.0 abcd	18.0 ab	5.8 abcd
		18.9 ABA	4.0 a	8.0 a	1.8 ab
		94.6 ABA	0.0 a	0.0 a	0.0 a

*Mean values followed by the same letters in columns do not differ at the significance level of *P* ≤ 0.05

For all explants and treatment combinations, the most frequent induction of adventitious bulbs was observed from explants derived from seedlings, although the differences between the mean values were not always significant. The induction of adventitious bulbs on the media containing 6% sucrose (alone or in combination with BAP) was significantly reduced as a result of chilling the explants beforehand. For the other combinations of culture media, there were no significant differences caused by chilling of the explants in the frequencies of bulb formation (Table 5). The number of bulbs per replicate was higher (although not always significantly) from explants cultured on treatments that cause higher-frequency callus formation.

Explants cultured with 6% sucrose (alone or in combination with BAP) formed several times fewer adventitious bulbs in comparison with non-chilled explants. Similar results were observed when explants were cultured with 3% sucrose, mainly for explants derived from the bulbs that developed from seedlings. There were no significant differences among treatments for explants treated with ABA in the number of bulbs obtained (0–6 bulbs) (Table 5).

### *Frequencies of adventitious bulb formation.*

Because of the higher frequencies of callus formation from explants derived from non-chilled bulbs treated with sucrose and BAP, these subsequent experiments were restricted to only two types of explants and four culture media.

**Figure 3:**
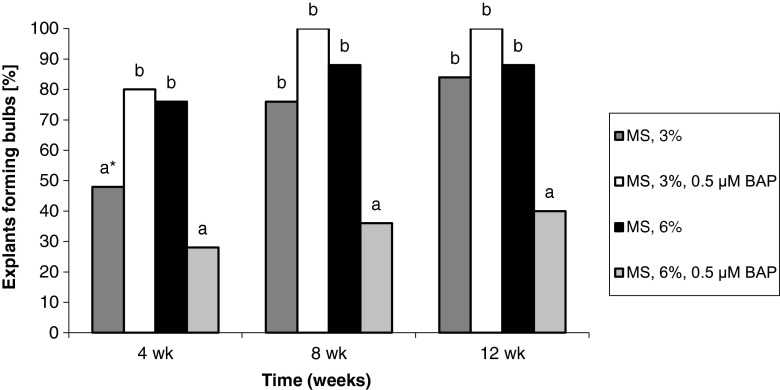
Effect of medium composition and time on the formation of bulbs from callus obtained from seedling-derived bulbs.

**Figure 4:**
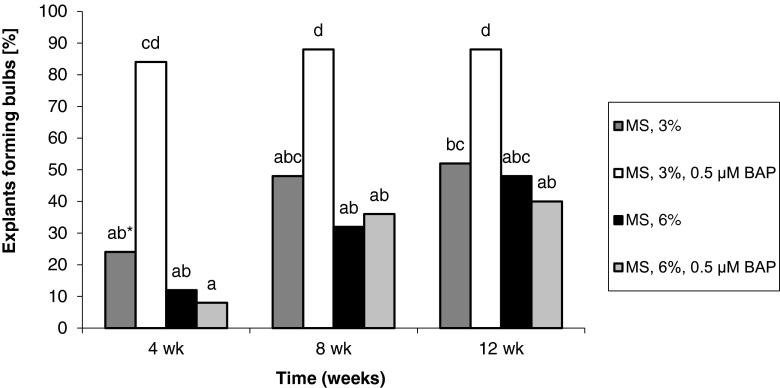
Effect of medium composition and time on the formation of bulbs from callus obtained from adventitious bulbs.

The percentage of explants forming adventitious bulbs gradually increased with increased length of culture. Bulb formation from seedling-derived callus tissue was promoted by the enrichment of the medium with 3% sucrose in conjunction with 0.5 μM BAP or with 6% sucrose alone. As a result, after 12 wk, the percentage of explants forming adventitious bulbs was 84–100%. Treatment with BAP and 6% sucrose significantly reduced (by more than half) the formation of adventitious bulbs (Fig. 3). For callus originating from adventitious bulbs, the highest percentage of explants form bulbs (84–88%) when treated with 3% and BAP. Means for other treatments ranged from 8 to 52% (Fig. 4).

**Figure 5:**
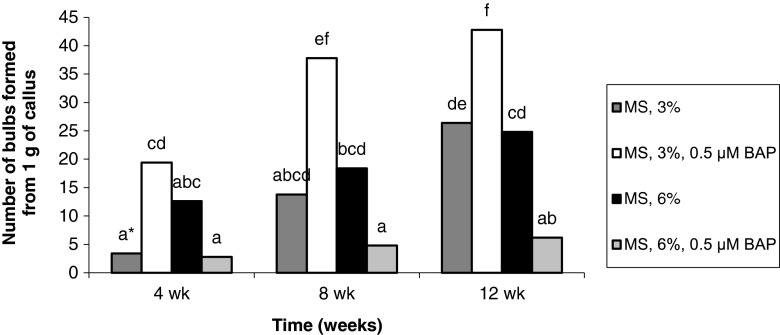
Effect of medium composition and time on the number of bulbs formed from callus obtained from seedling-derived bulbs.

**Figure 6:**
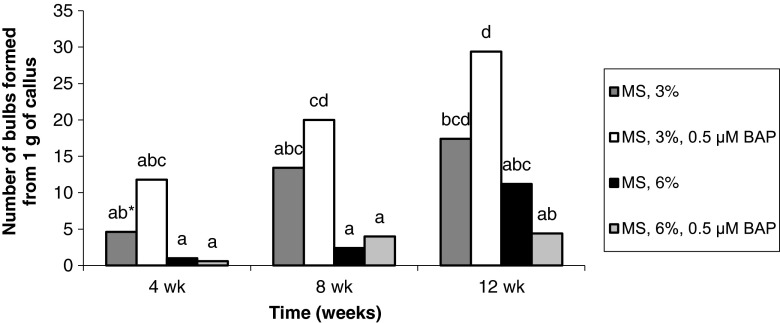
Effect of medium composition and time on the number of bulbs formed from callus obtained from adventitious bulbs.

For both types of explants, more bulbs formed on the media containing 3% sucrose and 0.5 μM BAP. Consequently, after 12 wk, 42.8 bulbs were obtained from callus originating from seedling-derived bulbs (Fig. 5) and 29.4 bulbs from the callus derived from adventitious bulbs (Fig. 6). The higher concentration of sucrose had a negative effect on the formation of bulbs in the case of callus derived from adventitious bulbs. Treatment with 6% sucrose resulted in the formation of 11.2 bulbs, and treatment with 6% sucrose and BAP resulted in the formation of 4.4 bulbs (Fig. 6).

### *Frequencies of callus tissue formation.*

**Figure 7:**
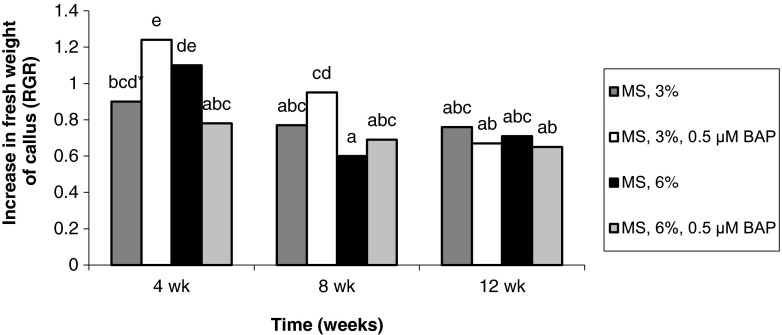
Effect of medium composition and time on the multiplication of callus obtained from seedling-derived bulbs.

**Figure 8:**
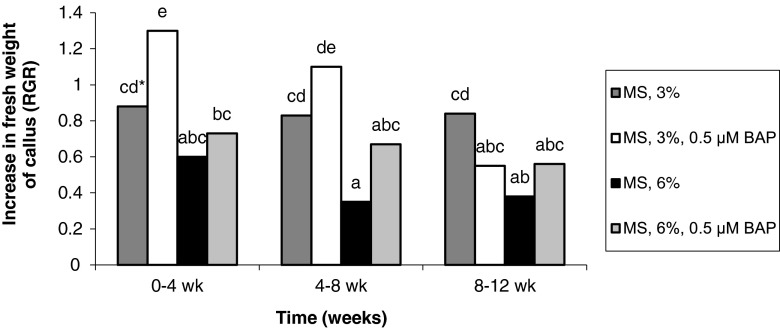
Effect of medium composition and time on the multiplication of callus obtained from adventitious bulbs.

The frequencies of callus formation from both types of explants gradually decreased over the culture period, although for treatments containing 6% sucrose, the frequency increased slightly in the last period (Figs. 7 and 8). The highest multiplication of callus (RGR > 1.2), regardless of the explant source, took place in the first month on medium containing 3% sucrose and 0.5 μM BAP (Figs. 7 and 8).

### *Anatomical observations.*

Adventitious bulbs obtained in the experiments had a meristem (Fig. 2*g*, *h*), which indicated that they had developed properly. The absence of a covering scale, which protects the bulb from desiccation, was probably due to high relative humidity in the Petri dish.

## Discussion

This study focused on the initial stages of *T. tarda* propagation *in vitro. In vitro* cultures of tulip are usually initiated from bulb scales (Nishiuchi 1980; Gude and Dijkema 1997), flower stems (Rice *et al.* 1983; Hulscher *et al.* 1992; Podwyszyńska and Marasek 2003) or ovaries (Ptak and Bach 2007). However, in the present study, cultures of *T. tarda* were initiated from seeds. Although using such explants was a more laborious and time-consuming, it was chosen to obtain more consistent and reliable starting material bulbs. Low-temperature seed treatment, used in this study, is required not only for proper growth and flowering (Rietveld *et al.* 2000), but also to break dormancy after sowing (Fortanier and Van Brenk 1975; Rouhi *et al.* 2010). The zygotic embryo in tulip seeds does not have a fully developed apical bud and thus needs to be chilled to break its dormancy and initiate further development (Niimi 1978). The chilling of seeds can promote germination in *T. tarda*. Gibberellic acid (GA) (Kudryavtseva 1980; Rouhi *et al.* 2010) can also be applied during cold stratification to break seed dormancy in *Tulipa kaufmanniana*. Famelaer *et al.* (2000) reported that cold-treated (4°C) seeds (embryos) were the most suitable explants for callus induction and proliferation of *Tulipa praestans*.

According to De Hertogh and Le Nard (1993), germination begins within a few days of seed hydration, as also confirmed with *T. kaufmanniana* (Rouhi *et al.* 2010). Cold-treated seeds of *Tulipa gesneriana* germinated after 5–6 wk (Famelaer *et al.* 1996). In this study, germination was not observed until 6 mo, as in nature. Nikolaeva (1977) claimed that *T. tarda*, apart from morphological dormancy, also underwent deep physiological dormancy, which explained its slow germination process. Furthermore, the germination of *T. tarda* took place under a 16-h photoperiod, while Custers and others (1992) observed that darkness was superior to 16-h light in producing stolons and bulblets during the formation of tulip seedlings.

The highest frequency of germination was observed from seeds cultured on 100% MS with 3% sucrose at 25°C. However, Boeken and Gutterman (1990) observed that *Tulipa systola* did not germinate at 25°C and germinated at 50% at 20°C. In *Lilium martagon*, the best germination rate was obtained from seeds cultured on media containing 50% MS with 1.5% sucrose at 20°C (Kędra and Bach 2005). Thus, optimum conditions for seed germination are different for various plants, even among the same genus.

The explants used in this research originated from bulbs derived from seedlings or from adventitious bulbs produced from callus tissue. There is lack of reports concerning such tulip explants, which is why we examined their utility for *T. tarda* propagation. Seedling-derived bulbs and adventitious bulblets were the most useful explants for callus initiation in *L. martagon* (Kędra and Bach 2005).

The morphogenic response of the explants depends on the presence and concentration of growth regulators in the medium (Gaspar *et al.* 2003). To initiate organogenesis in tulips, BAP is usually used, either alone or with auxin (Wright and Alderson 1980; Taeb and Alderson 1990; Minas 2007), although Famelaer *et al.* (1996) used only picloram and 2,4-dichlorophenoxyacetic acid (2,4-D) to obtain callus tissue. In this study, only 0.5 μM BAP was used to stimulate organogenesis. Explants treated with BAP usually formed undifferentiated, yellowish, loose callus tissue, from which small bulbs differentiated. Higher frequencies of callus formation were observed when explants were treated with sucrose, with or without BAP. Similarly, the induction of callus in bulblet scale cultures of wild growing *L. martagon* was stimulated by both 0.5 μM BAP and a growth regulator-free MS medium (Kędra and Bach 2005). In an experiment with ‘Apeldoorn’ tulips, bulb scale explants placed on an MS medium containing 2,4-D and kinetin also first produced callus and then organised structures (Baker *et al.* 1990). In bulblet scale cultures of *Lilium ledebourii*, the best media for adventitious bulblet formation contained 0.44 μM BAP with 0.54 μM naphthaleneacetic acid (NAA) (Bakhshaie *et al.* 2010). BAP with NAA was also used to form callus on scale explants in *Fritillaria imperialis* cultures (Witomska and Łukaszewska 1997). In bulb scale culture of *Muscari azureum*, the highest number of bulblets was obtained when treated with 0.5 μM BAP, together with 2,4-D and NAA (Uranbey *et al.* 2010).

In this study, adventitious bulbs appeared on the explants via direct or indirect (via callus tissue) organogenesis. In previous experiments, tulip bulbs had been obtained mainly from shoots that regenerated on initial explants (Rice *et al.* 1983; Bach and Ptak 2005). Ghaffor *et al.* (2004) observed the formation of adventitious tulip bulblets from embryogenic cultures treated with BAP and NAA. Direct *in vitro* organogenesis of bulblets, on an MS medium supplemented with NAA and BAP, was observed in the case of *Allium aflatunense* (Evenor *et al.* 1997) and *L. ledebourii* (Bakhshaie *et al.* 2010).

Induction of tulip bulbs can occur in darkness, at 5°C over a 10–12-wk period (Baker *et al.* 1990; Kuijpers and Langens-Gerrits 1997) when explants were cultured on an MS medium supplemented with 6% sucrose (Alderson and Taeb 1990; Famelaer *et al.* 1996). Our results demonstrate that chilling explants decrease the formation of callus and bulbs. The addition of 6% sucrose did not enhance, but sometimes even reduced, the number of bulblets obtained. Generally, the bulblets were forming on media containing 3% sucrose and 0.5 μM BAP in the dark.

In this study, the obtained bulbs did not have a tunic, perhaps because of the high relative humidity in the Petri dish. Such bulbs are vulnerable to drying out during cultivation *ex vitro*. In their experiment, Podwyszyńska and Ross (2003) also observed tulip bulbs without a tunic, or only partially covered.

ABA affects tissue development and maturation through regulation of gene expression during vegetative development of plants (Boonekamp 1997; Seo and Koshiba 2002). ABA stimulates the formation of bulbs (Kuijpers and Langens-Gerrits 1997) in hyacinth (Ii *et al.* 2002), lily (Kim *et al.* 1994) and garlic (Kim *et al.* 2003). The effects of ABA on the formation of tulip bulbs are less clear. Podwyszyńska and Ross (2003) claimed that ABA has an adverse effect upon tulip bulb formation, which we also observed. The formation of callus and bulblets on every explant was inhibited by ABA, particularly at higher concentrations. Similar conclusions have been drawn for gloriosa lily (Weryszko-Chmielewska and Kozak 2002).

Histological analyses of the obtained bulbs revealed the occurrence of bulb scales and meristem in the middle, which suggests proper bulb development. Famelaer *et al.* (1996) also observed a bulb-like structure with one or two smaller leaves and small centers of densely cytoplasmic cells at the base side of the bulblet. The formation of bulbs determines the efficiency of the micropropagation of tulip (Hulscher *et al.* 1992).

Based on our results, bulbs from seedlings and adventitious bulbs were suitable explants for propagating *T. tarda*. The process of adventitious bulb formation was promoted by sucrose and BAP and inhibited by ABA and chilling. Although there is still a need to further investigate culture conditions to enhance tulip proliferation and bulblet formation, our results demonstrate that the procedure presented in this report can be used for the multiplication of *T. tarda*.
